# Living labs for civic technologies: a case study. Community infrastructuring for a volunteer firefighting service

**DOI:** 10.3389/fpubh.2023.1189226

**Published:** 2023-05-12

**Authors:** Cristina Viano, Gregory Tsardanidis, Lorenzo Dorato, Alice Ruggeri, Alice Zanasi, Giannis Zgeras, Villy Mylona, Ioannis Efthymiou, Vasilis Vlachokyriakos

**Affiliations:** ^1^Department of Computer Science, University of Turin, Turin, Italy; ^2^Interuniversity Department of Regional and Urban Studies and Planning, University of Turin and Polytechnic of Turin, Turin, Italy; ^3^Open Lab Athens, Athens, Greece; ^4^School of Computing, Newcastle University, Newcastle, United Kingdom

**Keywords:** civic digital technologies, service co-production, community infrastructuring, civil protection, environmental protection, participatory design, urban living labs

## Abstract

Digital technologies are increasingly adopted and developed in living labs, to support and enable co-production processes around wellbeing related public services. This research report presents the case of one of the local laboratories established by the European project NLAB4CIT, in the city of Kaisariani, Greece. In order to enhance community engagement, participatory design methods are applied under an “infrastructuring” notion; the living lab model is reapproached as community infrastructure; and digital tools are understood as civic technologies. The article reports on the initial co-design phases, in order to provide other living labs on digital co-production with an overview on the socio-technical challenges encountered. These challenges concern how community actors can engage in the process of co-production of public services, and how digital technologies can be introduced to this purpose. Strength factors emerge such as the existence of an already active community, the creation of an enduring space of collaboration between researchers and citizens, and a civic approach to technology that makes them accessible and tailored. Open challenges concern the role of the public administration, the extent to which technologies are actually co-designed and co-developed, and some technical issues such as internet accessibility.

## 1. Introduction

All over Europe, various living lab (LL) initiatives are being implemented to address quality of life issues, by exploring digital solutions together with the concerned citizens. The European project NLAB4CIT^1^ aims to engage with citizens in the co-production of public services through digital technologies at local level, so that services are more responsive to emerging social needs. The project is expected to set up a network of Local Laboratories on civic technologies, starting from three laboratories in the cities of Collegno (Italy), Roeselare (Belgium), and Kaisariani (Greece).

The three laboratories have in common the focus on citizens’ active participation for maintaining and developing physical spaces, such as public gardens and parks (in Collegno), kindergartens (in Roeselare), and forests (in Kaisariani). These (peri)urban spaces provide communities with assets such as air quality, climate protection, and spaces for physical activities and where people can gather for social and cultural activities. They are an important resource contributing to the quality of life of urban inhabitants, and to their wellbeing. The latter is meant not only as physical and mental health, but also in terms of emotional and relational benefits coming from participation in the local community, and from access to services that are perceived respondent to one’s needs. This focus on a broader meaning of wellbeing is mirrored in the overall orientation of the NLAB4CIT labs. Like LLs in general, these labs emphasize multistakeholder co-production and digital innovation. However, the latter is functional to the former: digital tools are supporting or enabling instruments toward the restructuring of governance processes. Conversely, LLs on digital health and wellbeing are in most cases focused on the user-centered co-creation and testing of digital platforms and devices for the health system [see for instance (1–5)]. This article aims at complementing this kind of product-centered approach, offering insights on methodologies that address the community and dynamics in which the digital tools are introduced.

This article presents the Kaisariani Local Lab, on volunteer services for forest protection against fires. In this case, participatory design methods are applied under an “infrastructuring” notion (see section 2.1 for references). The LL is reapproached as community infrastructure. The research questions that guide this experimentation are (a) how stakeholders can engage in this local lab environment to co-produce public services, and (b) how digital technologies can be introduced to this purpose. This article reports on the socio-technical challenges encountered during the initial phases of the experiment, which is still ongoing. Section 2 of this article sets the theoretical background for the adopted methodologies. Section 3 describes the context, and the methodology adopted in the local lab. Results are presented in Section 4, and related socio-technical challenges discussed in section 5.

## 2. Background

### 2.1. Participatory design and infrastructures

Of relevance for LL approaches to citizen engagement, are the notions of “information infrastructures” and the practice of “infrastructure-ing.” The notion of infrastructure in Participatory Design (PD) refers to the substance rather than substrate of information systems (6), to make visible what remains unnoticed and in the background (7, 8). “Infrastructure-ing” is defined by Karasti et al. as an attempt to underline the processual, ongoing quality of such participatory activities, and can be a way of advancing community interests through integrating design activities for extended periods within local communities (9, 10). Such activities, instead of focusing on the target artifact or service, are mainly concerned with its situated and contextual socio-political parameters (11).

Of particular relevance to our work, is the work of the Malmö Living Labs (12, 13). Here, infrastructuring is construed as a long-term platform for social innovation. Through embedded LLs, relationships and engagements are developed with local communities. Similarly, in past related work (14), the authors developed an approach for the emergence of community solidarity practices and methods for infrastructuring social innovation (15). The authors bring the focus on the negotiations (and agonistic processes) that took place between researchers, local communities, and other civic actors; and on participatory methods and strategies that create the conditions for civic-led co-production. The project reported in this article is an attempt to further unpack the complexities at play in these negotiations.

### 2.2. (Urban) living labs as community infrastructures

Living labs (LLs) are one of the most common approaches through which community infrastructures take place. LLs are broadly defined as real-life test and experimentation environments and ecosystems (16), and as physical regions or virtual realities (17) for the creation, testing, and validation of new products, services, and technologies (18). Major networks and international initiatives, such as the European Network of Living Labs (ENOLL), identify five key components: (a) active user involvement, (b) co-creation approach, (c) real-life settings, (d) multi-stakeholder participation, and (f) a multi-method approach (19, 20). For an extensive overview of the concept of LL and its origin and paradigms, we refer to a review by Hossain et al. (21). LLs overlap with other types of collaborative innovation where the public plays an important role, such as innovation labs (18, 22) and policy labs (23).

Another definition is that of Urban Living Labs (ULLs) (24, 25). This resonates with the understanding of community and information infrastructure mentioned above, due to the focus on socio-political contexts and processes. Their core components are: (a) geographical embeddedness; (b) intentional ongoing evaluation and learning, through municipalities-researchers partnerships; (c) citizens participation; and (d) alternative modes of leadership to those of the private sector and traditional urban planning (26, 27). Rather than focusing on digital technologies and their users, the attention is on change in governance and policies, on geographical situatedness (28), and on the active role of citizens (29).

### 2.3. Digital co-production

In this project, our attempts to establish a local LL, as an infrastructure for the community, aims to create the conditions for digital co-production. Scholars in policy analysis and public management (30–33) understand digital co-production as the collaboration between citizens, government and other actors, improved, supported or enabled by digital tools in the different stages of service delivery. However, it is observed that participation is often limited to the design and monitoring of the service, and to the “citizen-sourcing” (30) mode of collaboration between citizens and government, rather than contributing to the actual delivery of services (30, 34, 35). The same happens with ULLs, due to their experimental nature (34).

In order to address these limits, useful approaches come from recent works on co-production, in fields such as Human-Centered Design (HCD) and Human-Computer Interaction (HCI). The recent civic turn in such fields [e.g., (20, 36–38)] has seen a proliferation of research concerned with developing socio-technical tools and processes aiming to support dialogs and collaborations between civics and between civics and institutions. Research in this area is motivated by aspirations to advance equitable societies through fostering civic engagement, in areas such as, among others, local politics (39, 40), social innovation (12, 15, 41), and grassroots civic advocacy initiatives (14, 39, 42, 43).

## 3. Context and methods

### 3.1. The NLAB4CIT local labs and the Kaisarieni context

The three local labs of the NLAB4CIT project combine the concept of digitally enabled co-production (30–33) and of digital civic technologies (32, 41, 44). The labs are defined as local in order to focus on their geographical, social and political situatedness, as in ULLs. The innovation processes revolve around the co-production of services of public interest, rather than on market-oriented improvement of digital products. Services can either be provided by the public administrations, or by the civil society supported in different ways by the public actor. A broad understanding of “lab” is adopted, acknowledging that the modes through which civic technologies are introduced can vary a great deal. There are no pre-defined common governance models across the three labs. Civic digital technologies are expected to facilitate citizens’ participation in collective forms, rather than just digitizing services for efficiency purposes. Intentional actions are taken throughout the whole cycle of design, development, and use of the digital tools, in order to embed public values (e.g., openness, inclusion, accessibility, and technological sovereignty) in their features.

The Greek local lab has been activated in the municipality of Kaisariani, a suburb of Athens identified as a left-wing stronghold since its historic role in the Greek resistance during World War II. This background has forged a common identity of active citizen participation and community organization, which also caused the Skopeftirio Park green area in the Hymettus Mountain to come under public ownership. In this context, the Volunteer Forest Protection Team of Kaisariani (VFPTK) was born. VFPTK is a self-organized team of volunteer citizens (70–120 persons) to protect the Hymettus forest against fires. After a series of interviews with the municipality civil servants about possible sectors and initiatives related to community wellbeing, in which intervention could take place, VFPTK was selected as the main pilot case scenario, due to their important contribution to the community and their technical needs.

### 3.2. Community engagement and co-design

Following an infrastructuring approach for participatory design (9), and related methods and strategies for participatory action research in the field of digital civic co-production (14, 15), the research teams set up a lab in Kaisariani. The research team engaged with the VFPTK, starting with preparatory meetings to delve into: (a) their activities and the status of their existing technical infrastructure, (b) organizational models developed over the years, and (c) problems and challenges, which helped map potential interventions. These meetings were in the form of field visits in VFPTK facilities and outposts. The research team kept notes on explanations, created diagrams and took photos.

**Figure 1:**
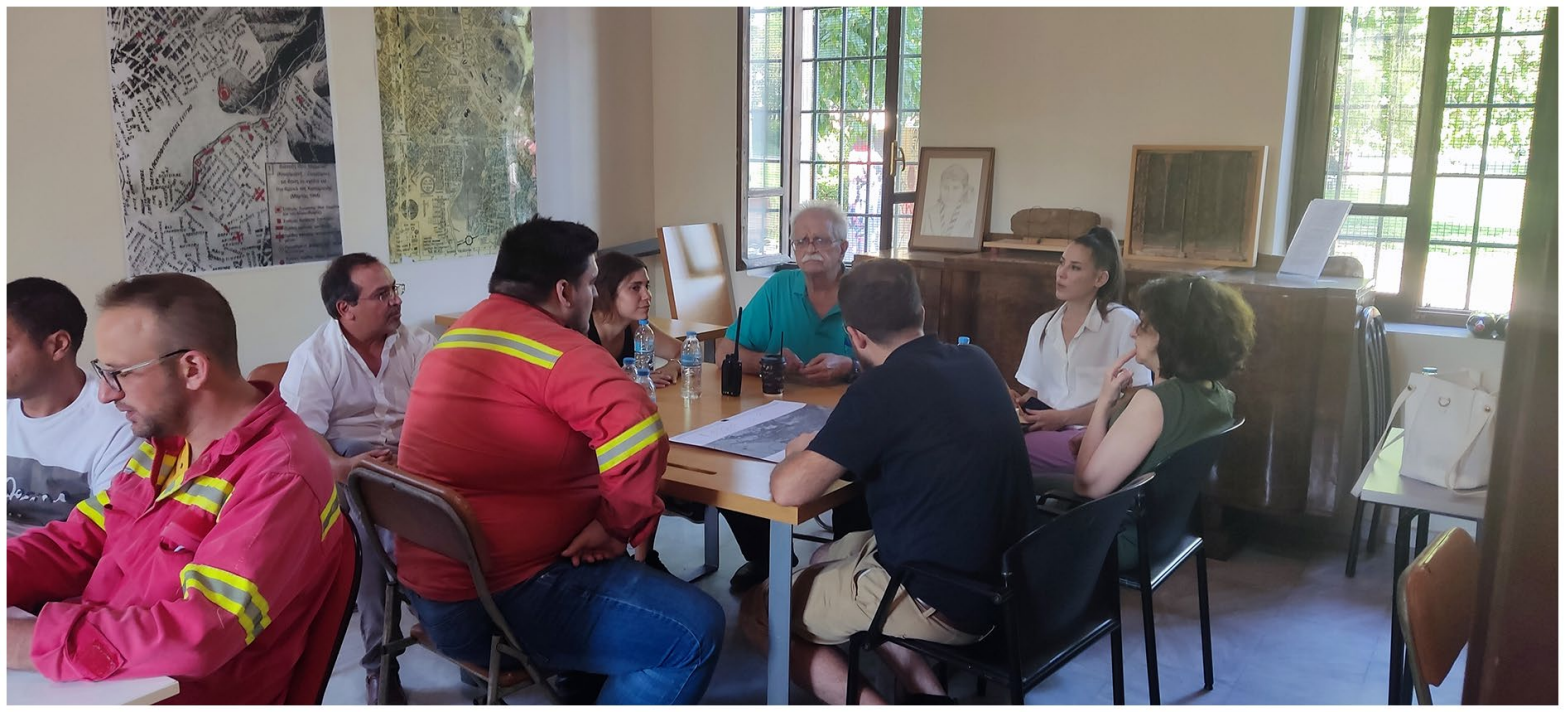
Co-design Workshop, 5.6.2022.

In the second phase, a series of co-design sessions was organized, in order to identify specific challenges in the everyday activities and to co-design technology-enabled solutions. VFPTK members, employees of the Municipality of Kaisariani and research team members worked together. Co-design canvases (available as supplementary materials to this article) were used to stimulate and document the discussion. Researchers undertook facilitating roles, while the VFPTK members were the main contributors in information and ideas. The main workshop took place at the Kaisariani Museum of National Resistance on the June 5, 2022, and lasted 3 h. There were six members of VFPTK, five municipal employees (three from IT department, one from the civil protection service, and one from the administration), and three researchers. Participants were split into two groups of seven, both representing all stakeholders. Data were collected through the co-design canvases and researchers’ notes (Figure 1).

**Figure 2:**
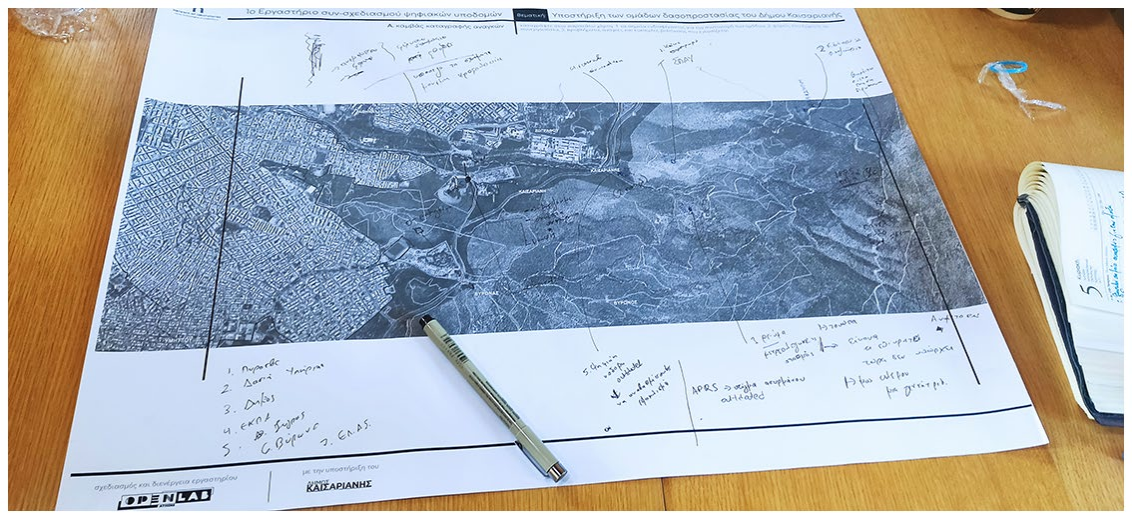
Canvas 1 filled at the Co-design Workshop of 5.6.2022.

During the first part of the co-design workshop participants mapped places of interest in the area (e.g., fire-fighting headquarters, watchtower) and problems regarding these spaces. An aerial photography of the area was used. This canvas format was selected because from preliminary meetings it was evident that challenges and potential interventions were related to specific locations (Figure 2).

In the second part, participants worked on service proposition canvases. They were required to elicit: the needs the proposed infrastructure will address, its desired functionalities, and challenges that may arise. Finally, they had to draw a detailed diagram of the desired infrastructure in an appropriate form (e.g., a flow chart and data structure or blueprint). Researchers played the role of facilitators. Four services were co-designed with a high level of detail (two by each team). All data collected through the canvases were analyzed by the research team and restructured as independent reports, containing the service details and which technologies can be used to produce them. These reports were submitted to VFPTK, which internally discussed them and concluded to start implantation of three distinct actions (see section 4.2).

In the third phase, we focused on actually co-producing the services along with the citizens. For the first service (see section 4.2), the equipment required was immediately provided and VFPTK set up the service. For the other more complex services (see section 4.2), researchers produced detailed technical reports that were later discussed in collaborative sessions with the VFPTK to finalize the desired functionality. This is an ongoing process in which both researchers and VFPTK contribute with their respective knowledge and technical capacity. Open-source software and open design hardware are used as much as possible. The results of these actions are reported below.

## 4. Results

### 4.1. Existing infrastructure

**Figure 3:**
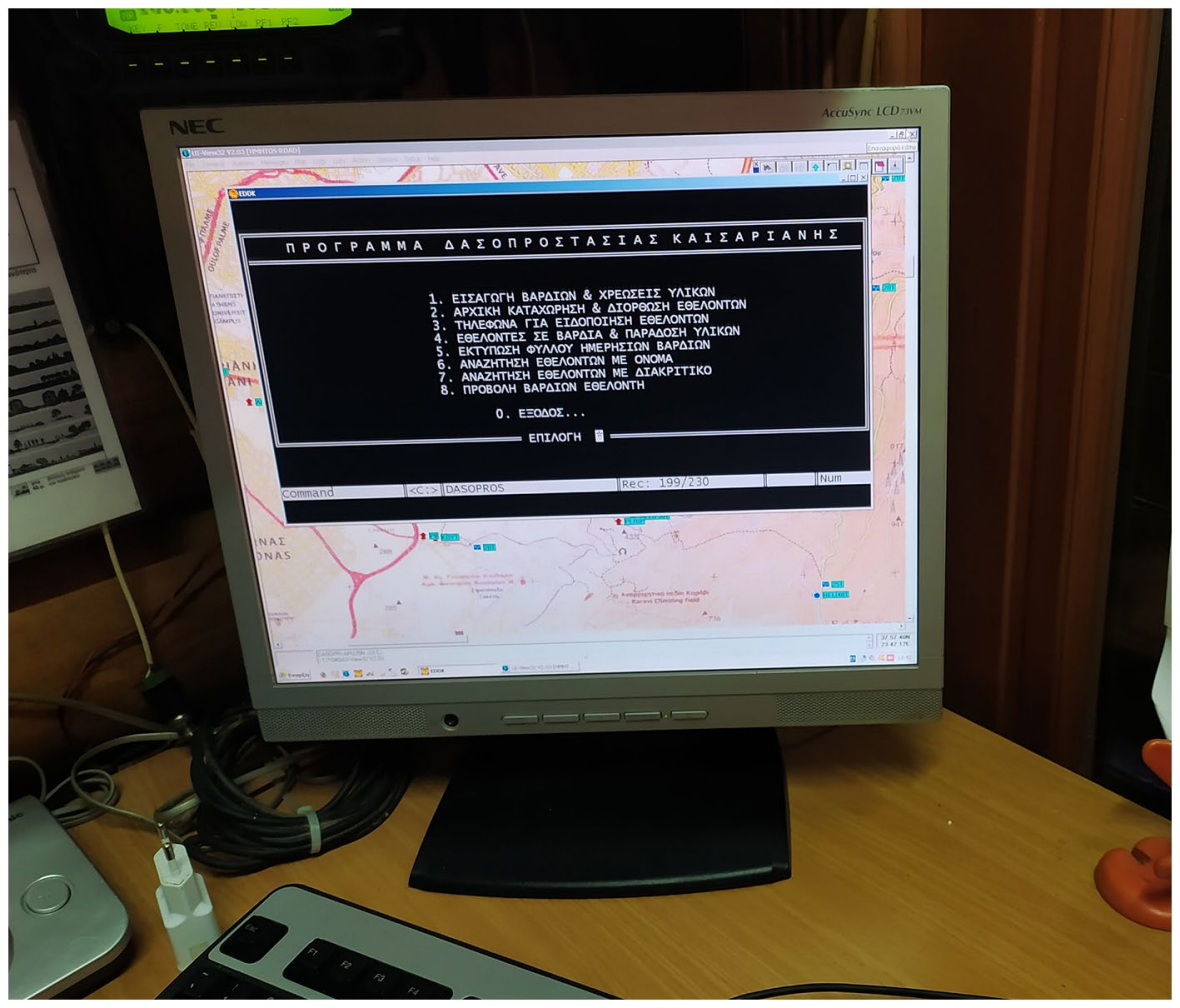
The “homebrew” database of VFPTK.

Volunteer Forest Protection Team of Kaisariani existing infrastructure has been developed over the years by VFPTK members and is composed of *“*homebrew*”* systems. These systems use mostly unconventional or outdated practices and methods, selected more for the ability of VFPTK members to employ them than on grounds of adequacy. Nevertheless, their usage over a long period of time and constant small-scale improvements made them tailored to the needs of the team. These systems include a very high frequency (VHF) radio station, a do-it-yourself weather station and a *h*omebrew local database (45) built on dBASE and running on Windows XP or older operating systems (Figure 3).

The database was built from scratch by one of the most long-standing members, without professional experience of programming. The system includes most of the functionalities needed to administer VFPTK, such as a member’s registry and shift management.

Volunteer Forest Protection Team of Kaisariani has been working as an improvised technology development lab for decades. The group benefits from the skills of amateur radio broadcasters, amateur programs, and professional technicians. The municipality supports by providing the space for their headquarters and by occasionally repairing their vehicles, but no consistent direct funding is made available. Self-funding from sympathizers is necessary. VFPTK preserves a strong do-it-yourself and maintenance culture. Technical equipment, from PCs to vehicles, are not easily replaceable: the team tries to maintain everything as much as possible, even if this compromises the usefulness, ease-of-use, and reliability.

### 4.2. Co-designed services

Through co-design, the participants developed three distinct services, mostly replacements of or enhancements to existing ones:

1. A device to record operational data transmitted through the VHF radio.
2. A weather station.
3. A software system for the team’s management.

The first system was rapidly implemented, since the volunteers had already decided on the device they needed to record data. A commercial solution was purchased with the project funding, and added to the existing VHF radio station by VFPTK members.

**Figure 4:**
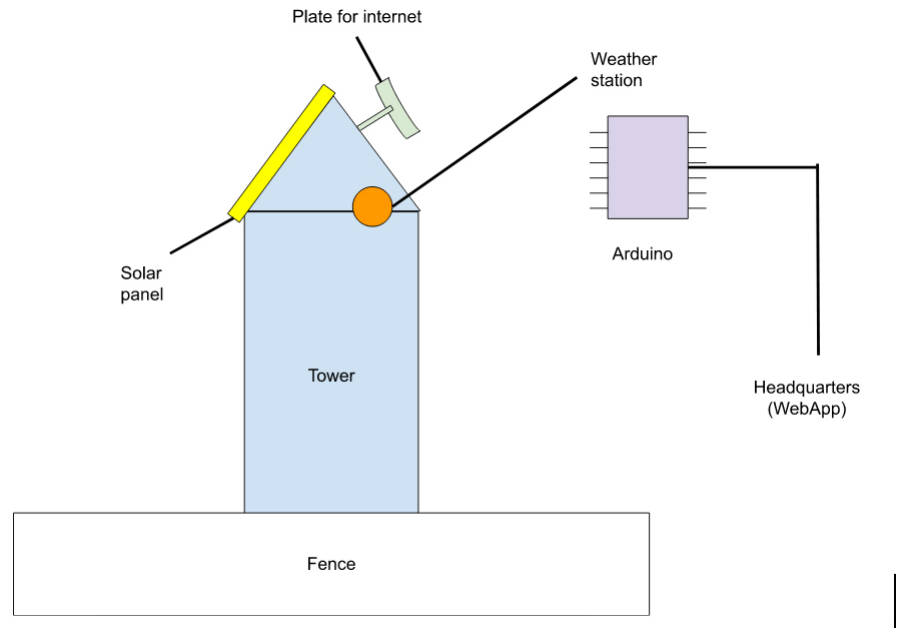
Weather station diagram created on Canvas 2 at the Co-design workshop of 5.6.2022.

The second service is a weather station to be used for gathering data about temperature, humidity, wind, and atmospheric pressure. It was designed in detail at the co-design workshop (Figure 4).

The station will be positioned in one of the watchtowers operated by VFPTK. It will use (Arduino) open hardware microcomputers and will transmit data to the headquarters *via* the internet. Arduino was selected for its simplicity, and since one of the volunteers has relevant expertise for developing and maintaining the service. Through the research project, the Municipality of Kaisariani has funded the installation of solar panels to provide energy, and has hired the mentioned volunteer with Arduino expertise, to help co-develop the weather station with VFPTK members. Serious obstacles remain, such as internet access in the remote watchtower.

The third service aims to totally replace the homebrew local database. The new system will have all the functionalities of the old one (member’s registry, shift management), and will also support a vehicle registry. It will be web based and will follow a three-tier architecture. This way, VFPTK administrators will be able to use the system at their headquarters as well as in the main summer outpost. Until now, they have been using USB storage devices to move data from one local database to others, which caused several problems. A crucial aspect is the addition of a mobile phone application through which volunteers will be able to register for shifts and receive push notifications. The research team prepared a detailed technical report, then discussed extensively with VFPTK members to get feedback. In this case, development will be undertaken mainly by the research team using open-source web technologies such as Angular and the Strapi content management system (CMS), since VFPTK does not have these advanced capabilities. A prototype of the member’s registry has already been developed in Strapi. The goal is to have a beta implementation of the system in the beginning of summer 2023.

### 4.3. Community engagement

The volunteers engaged in the process were already part of a highly committed group, willing to provide satisfactory solutions to practical problems. Their involvement and intense interaction over a long period of time have formed a strong community, able to overcome any obstacles with limited external support. Personal skills, both professional and amateurial, were put at disposal of the group. The same now happens in the co-production process. The latter was regarded by team members as a long-awaited opportunity for change, especially for younger members. But even older members overcame their reluctance, which was rooted in previous unsuccessful attempts to update systems.

Previous commitment of public institutions was limited. VFPTK complained about inadequate and irregular support from local, regional, and national public authorities. The engagement of Kaisariani Municipality has intensified during the co-production process. An example is the allocation of public funds for both material and human resources of the second co-designed service.

The constant engagement of the research team, along with the project resources available, played an important role. As regard the first service, the acquisition of a commercial technology, even though diverging from the co-design methodology, was crucial in proving to VFPTK the usefulness of the process, and thus helped gain their trust. The co-design intervention on the second services facilitated the coming together of the different stakeholders. As the co-production process continues, it will be possible to see how different stakeholders contribute to overcome remaining obstacles, such as internet access. For the third service, the research team plays a more relevant role in technology development: coding the system together with the volunteers is not possible at the moment due to the required skills, but VFPTK decided on the data structure and user flow.

## 5. Discussion

This section discusses the results presented above, highlighting the socio-technical challenges arisen (strengths and criticalities), and addressing the two research questions concerning (a) how actors engage in the lab, and (b) how civic digital technologies can be introduced.

The relevance of specific interventions in support of VFPTK’s services, through digital tools, was recognized by the researchers for the following reasons. Firstly, the field of forest protection, already addressed for a long time by citizens concerned for Skopeftirio Park, has become more important in recent years because of the acceleration of climate change. Secondly, many needs were related to the lack or inadequacy of technical infrastructures, which affected the effective coordination of the actors. Moreover, the opportunity to combine outdated equipements with more advanced civic technologies, and the availability of additional resources from European funds, encouraged the actors to participate.

The following strengths emerged. The existence of an active community was an important precondition for getting *actors engaged in the co-production process*. The rich experience of community culture should not be disregarded but cherished and incorporated in any community infrastructuring project. Moreover, a common space has emerged where researchers and citizens share knowledge and experience, working as equals. Resistance to change, which is common when such digital infrastructures are introduced, can be overcome when a circle of trust is created, and community members understand that the research team has a long-term commitment to producing positive effects with mutual interest. As regards the introduction of *digital tools*, it is worth noting that the educational part of engaging citizens with civic technologies is integrated in an extended co-design and co-development phase, where technology is discussed as tangible, accessible, and suitable to addressing the challenge of forest protection. The attitude of the volunteers (do-it-yourself and maintenance) to technical devices is also relevant. The second service is a good empirical case of selecting simple and open digital solutions on which expertise is already available, and of motivating different actors to cooperate.

Some criticalities were observed. Regarding *actors engagement*, the role of public administration in the case study deserves attention. Informal LLs such as VFPTK have been producing civic technology for years because they have been disconnected from public institutions. The research project has been an opportunity for the municipality to engage and acknowledge the needs of the community. Regarding the *digital tools*, firstly, the digital systems implemented with civic effort mostly involve unconventional practices and methods. This can strengthen the willingness of members to find accessible solutions, but these solutions could be selected more for the ability of members to employ them rather than for their adequacy. Secondly, participants must be aware of how deeply the community can actually influence the technology. In the first service, the community wanted to directly adopt a commercial technology that could “do the job,” and did not share the researchers’ academically-motivated considerations on co-design. Similarly, an open issue concerns to what extent technology experts and communities can co-develop digital tools. A deep involvement is possible when the community has relevant expertise, as with the second service. But often, the research team needs to do the bulk of the development work, as with the third service. Thirdly, any digitally-enabled co-production process must address technical limitations. In the example of the second service, there is currently no internet access in the watchtower, neither from cable internet nor from the weak mobile signal. It will be necessary to find innovative solutions along with the communities, since the telecommunication companies contacted are unwilling to cooperate. One option is to get internet access *via* a direct link from the nearby University of Athens campus.

## 6. Conclusion

The Kaisariani local lab adopts an infrastructuring approach and participatory methods that create the conditions for services co-production, supported by civic technologies. This article reports and discusses the sociotechnical challenges emerged during the first phases of the lab implementation. The reported observations have some limits in that the Kaisariani lab is ongoing, and some issues are still open. The results attain in particular the initial phases of a LL on civic technologies: namely, the engagement of the actors within the local community, the analysis of the digitally-supported services, and the design of new digital tools. In this regard, these insights are relevant for LLs on digital coproduction of wellbeing-related services.

The applied methodology seems effective in establishing trust; creating a common space for pooling knowledge and resources; making digital technologies tangible, accessible, and tailored to community needs. However, contextual factors such as the volunteers’ commitment and their attitude to technologies have been core preconditions for the collaborative process. Open issues concern the long-lasting commitment of the public administration, some technical limitations, and the extent to which the community actually wants and can be active in co-designing and co-developing digital tools. The processes of bringing actors together, and of introducing technologies with a civic approach, are strictly intertwined. Cultural, social, and political preconditions had an influence on the setting up of the lab. Conversely, the co-design of open and customizable technologies activates collaborations and resources in the community.

## Data availability statement

The raw data supporting the conclusions of this article will be made available by the authors, without undue reservation.

## Ethics statement

Ethical review and approval was not required for the study on human participants in accordance with the local legislation and institutional requirements. The patients/participants provided their written informed consent to participate in this study. Written informed consent was obtained from the individual(s) for the publication of any potentially identifiable images or data included in this article.

## Author contributions

AZ and CV wrote the first draft. GT, GZ, IE, and VM implemented the Kaisariani lab and wrote sections 1, 2.1, 2.3, 3 and 4. CV, LD and AR wrote sections 1, 2.2, 5 and 6. VV wrote parts of the background work sections 2.1, 2.2 and 2.3 and made overarching changes in relation to civic technology literature and community infrastructure-ing. All authors contributed to the manuscript, critically reviewed its content, and have read and agreed to the published version of the manuscript.

## Funding

The NLAB4CIT project is funded by the European Commission, Grant Agreement number LC-01688130.

## Conflict of interest

The authors declare that the research was conducted in the absence of any commercial or financial relationships that could be construed as a potential conflict of interest.

## Publisher’s note

All claims expressed in this article are solely those of the authors and do not necessarily represent those of their affiliated organizations, or those of the publisher, the editors and the reviewers. Any product that may be evaluated in this article, or claim that may be made by its manufacturer, is not guaranteed or endorsed by the publisher.

## Footnotes

^1^*Network of Laboratories for Civic Technologies Co-Production: Digital Services for the Public Administrations of the Future,* www.nlab4cit.eu. *Funded under the Preparatory Action: Smart local administration using IoT, AI, VR and Machine Learning tools to get closer and more present to the citizen* (CNECT/2020/3855995), launched in July 2020 by the European Commission Directorate-General for Communications Networks, Content and Technology.

## References

[1] Bernaerts S De Witte NAJ Van der Auwera V Bonroy B Muraru L Bamidis P. Rehabilitation supported by technology: protocol for an international cocreation and user experience study. JMIR Res Protoc. (2022) 11:e34537. doi: 10.2196/34537, PMID: 35266874 PMC8949709

[2] Burrows R Mendoza A Pedell S Sterling L Miller T Lopez-Lorca A. Technology for societal change: evaluating a mobile app addressing the emotional needs of people experiencing homelessness. Health Inform J. (2022) 28:146045822211467. doi: 10.1177/14604582221146720, PMID: 36548199

[3] Dietrich T Guldager JD Lyk P Vallentin-Holbech L Rundle-Thiele S Majgaard G. Co-creating virtual reality interventions for alcohol prevention: living lab vs. Front Public Health. (2021) 9:634102. doi: 10.3389/fpubh.2021.634102, PMID: 33791269 PMC8005569

[4] Fotis T Kioskli K Sundaralingam A Fasihi A Mouratidis H. Co-creation in a digital health living lab: a case study. Front Public Health. (2023) 10:892930. doi: 10.3389/fpubh.2022.892930, PMID: 36733280 PMC9887018

[5] Martinez S Berkås S Fensli R. Agder living lab: co-creation of inclusive health solutions for and with citizens. Int J Integr Care. (2016) 16:1–2. doi: 10.5334/ijic.2580

[6] Neumann L.J. Star S.L. (1996). “Making infrastructure: the dream of a common language.” in PDC. 231–240.

[7] Karasti H. (2014). “Infrastructuring in participatory design.” in *Proceedings of the 13th Participatory Design Conference: Research Papers-Volume 1*. 141–150.

[8] Star SL. The ethnography of infrastructure. Am Behav Sci. (1999) 43:377391

[9] Karasti H Baker KS Halkola E. Enriching the notion of data curation in e-science: data managing and information infrastructuring in the long term ecological research (LTER) network. Comput Support Cooperat Work. (2006) 15:321–58. doi: 10.1007/s10606-006-9023-2

[10] Karasti H. Syrjänen A. (2004). “Artful infrastructuring in two cases of community PD” in *Proceedings of the Eighth Conference on Participatory Design: Artful Integration: Interweaving Media, Materials and Practices*. 1, 20–30.

[11] Volkmar PV Wulf V. Infrastructuring: toward an integrated perspective on the design and use of information technology. J Assoc Inf Syst. (2009) 10:447. doi: 10.17705/1jais.00195

[12] Björgvinsson E. Ehn P. Hillgren P. (2010). “Participatory design and democratizing innovation.” in *Proceedings of the 11th Biennial participatory design conference*, 41–50.

[13] Björgvinsson E Ehn P Hillgren P. Agonistic participatory design: working with marginalised social movements. CoDesign. (2012) 8:127–44. doi: 10.1080/15710882.2012.672577

[14] Vlachokyriakos V. Crivellaro C. Wright P. Karamagioli E. Staiou E.-R. Gouscos D.. (2017). “HCI, solidarity movements and the solidarity economy” in *Proceedings of the 2017 CHI Conference on Human Factors in Computing Systems*. 3126–3137.

[15] Vlachokyriakos V. Crivellaro C. Wright P. Olivier P. (2018). “Infrastructuring the solidarity economy: unpacking strategies and tactics in designing social innovation” in *Proceedings of the 2018 CHI Conference on Human Factors in Computing Systems*, 1–12.

[16] Ballon P Schuurman D. Living Labs: Concepts, Tools and Cases, *vol. 17*. London: Emerald Group Publishing Limited, (2015).

[17] Leminen S. Coordination and participation in living lab networks. Technol Innov Manag Rev. (2013) 3:5–14. doi: 10.22215/timreview/740

[18] Schuurman D. Tõnurist P. (2017). Innovation in the Public Sector: Exploring the Characteristics and Potential of Living Labs and Innovation Labs. Technol Innov Manag Rev 7, 7–14. doi: 10.22215/timreview/1045

[19] Robles A.G. Hirvikoski T. Schuurman D. Stokes L. (2016). Introducing ENoLL and its living lab community. ©ENoLL Available at: https://issuu.com/enoll/docs/enoll-print

[20] Boehner K. DiSalvo C., (2016). “Data, design and civics: an exploratory study of civic tech.” in *Proceedings of the 2016 CHI Conference on Human Factors in Computing Systems (CHI '16). Association for Computing Machinery*, New York, NY, USA. 2970–2981.

[21] Hossain M Leminen S Westerlund M. A systematic review of living lab literature. J Clean Prod. (2019) 213:976–88. doi: 10.1016/j.jclepro.2018.12.257

[22] McGann M Wells T Blomkamp E. Innovation labs and co-production in public problem solving. Public Manag Rev. (2021) 23:297–316. doi: 10.1080/14719037.2019.1699946

[23] Lewis JM McGann M Blomkamp E. When design meets power: design thinking, innovation, and the politics of policymaking. Policy Polit. (2019). doi: 10.1332/030557319X15579230420081

[24] Aernouts N Cognetti F Maranghi E. Urban living lab for local regeneration In: Beyond Participation in Large-scale Social Housing Estates. Cham: Springer (2023)

[25] Marvin S Bulkeley H Mai L McCormick K Voytenko Palgan Y. Urban Living Labs Experimenting With City Futures. New York: Routledge (2018).

[26] Bulkeley H Coenen L Frantzeskaki N Hartmann C Kronsell A Mai L. Urban living labs: governing urban sustainability transitions. Curr Opin Environ Sustain. (2016) 22:13–7. doi: 10.1016/j.cosust.2017.02.003

[27] Bulkeley H Marvin S Palgan YV McCormick K Breitfuss-Loidl M Mai L. Urban living laboratories: conducting the experimental city? Eur Urban Reg Stud. (2019) 26:317–35. doi: 10.1177/0969776418787222

[28] Karvonen A van Heur B. Urban Laboratories: experiments in reworking cities: introduction. Int J Urban Reg Res. (2014) 38:379–92. doi: 10.1111/1468-2427.12075

[29] Brandsen T Honingh M. Definitions of co-production and co-creation In: Brandsen T Verschuere B Steen T, editors. Co-Production and Co-Creation. Engaging Citizens in Public Services. New York: Routledge (2018). 9–17.

[30] Linders D. From e-government to we-government: defining a typology for citizen coproduction in the age of social media. Gov Inf Q. (2012) 29:446–54. doi: 10.1016/j.giq.2012.06.003

[31] Lember V. The increasing role of digital technologies in co-production and co-creation In: Brandsen T Verschuere B Steen T, editors. Co-Production and Co-Creation. Engaging Citizens in Public Services. New York: Routledge (2018). 115–27.

[32] Schrock A. R. (2019). What is civic tech? Defining a practice of technical pluralism in The Right to the Smart City. (eds.) Cardullo P. Feliciantonio C. Di Kitchin R. (London: Emerald Publishing Limited), 125–133

[33] Yuan Q. (2019). “Co-production of public service and information technology: a literature review” in *Proceedings of the 20th Annual International Conference on Digital Government Research*, 123–132.

[34] Nesti G. Co-production for innovation: the urban living lab experience. Polic Soc. (2018) 37:310–25. doi: 10.1080/14494035.2017.1374692

[35] Clifton J Fuentes DD García GL. ICT-enabled co-production of public services: Barriers and enablers. A systematic review. Inf. Polity. (2020) 25:25–48. doi: 10.3233/IP-190122

[36] Crivellaro C. Taylor A. Vlachokyriakos V. Comber R. Nissen B. Wright P. (2016). “Re-making places: HCI, «community building» and change.” in *Proceedings of the 2016 CHI Conference on Human Factors in Computing Systems*, 2958–2969.

[37] Satchell C. Dourish P. (2009). “Beyond the user: use and non-use in HCI.” in *Proceedings of the 21st annual conference of the Australian computer-human interaction special interest group: Design: Open 24/7 (OZCHI '09)*. Association for Computing Machinery, New York, NY, USA, 9–16.

[38] Gordon E D’Ignazio C Mugar G Mihailidis P. (2013) Civic Media Art and Practice: Toward a Pedagogy for Civic Design. Interactions (2017) 24:66–69. doi: 10.1145/3041764

[39] Asad M. Le Dantec C.A. (2015). “Illegitimate Civic Participation: Supporting Community Activists on the Ground.” in *CSCW 2015: Proceedings of the ACM 2015 conference on Computer supported cooperative work*, New York, NY, USA, ACM. 1694–1703.

[40] Dimond J.P. Dye M. LaRose D. Bruckman A.S. (2013) “Hollaback!: the role of storytelling online in a social movement organization” in *Proceedings of the 2013 conference on Computer supported cooperative work*. 477–490.

[41] Saldivar J Parra C Alcaraz M Arteta L Cernuzzi L. Civic Technology for Social Innovation. Comput Supported Coop Work. (2019) 28:169–207. doi: 10.1007/s10606-018-9311-7

[42] Puussaar A Johnson IG Montague K James P Wright P. Making open data work for civic advocacy. Proc ACM Hum Comput Interact. (2018) 2:143. doi: 10.1145/3274412

[43] Maskell T Crivellaro C Anderson R Nappey T Araújo-Soares V Montague K. “Spokespeople: Exploring routes to action through citizen-generated data.” in Proceedings of the 2018 CHI Conference on Human Factors in Computing Systems, (2018) 1–12.

[44] Certomà C Corsini F. Digitally-enabled social innovation. Mapping discourses on an emergent social technology. Innovation. (2021) 34:560–84. doi: 10.1080/13511610.2021.1937069

[45] Voida A. Harmon E. Al-Ani B. (2011). “Homebrew databases: complexities of everyday information management in nonprofit organizations” in *Proceedings of the SIGCHI Conference on Human Factors in Computing Systems*. 915–924.

