# Supplementary material for: Living labs for civic technologies: a case study. Community infrastructuring for a volunteer firefighting service
Source: Front Public Health. 2023 May 12;11:1189226. doi: 10.3389/fpubh.2023.1189226 (PMC10213287; doi:10.3389/fpubh.2023.1189226)
Supplement: Supplementary file 1 [file Data_Sheet_1.PDF]

Network of laboratories
Civic technologies co-production

Workshop: Co-designing digital infrastructures

thematic aera : **Supporting forest protection teams of Municipality of Kesariani**

A. Mapping needs canvas

map in the following map: 1. points of interest for team's coordination, 2. institution with which you cooperate , 3.problems, needs and opportunities of improvement

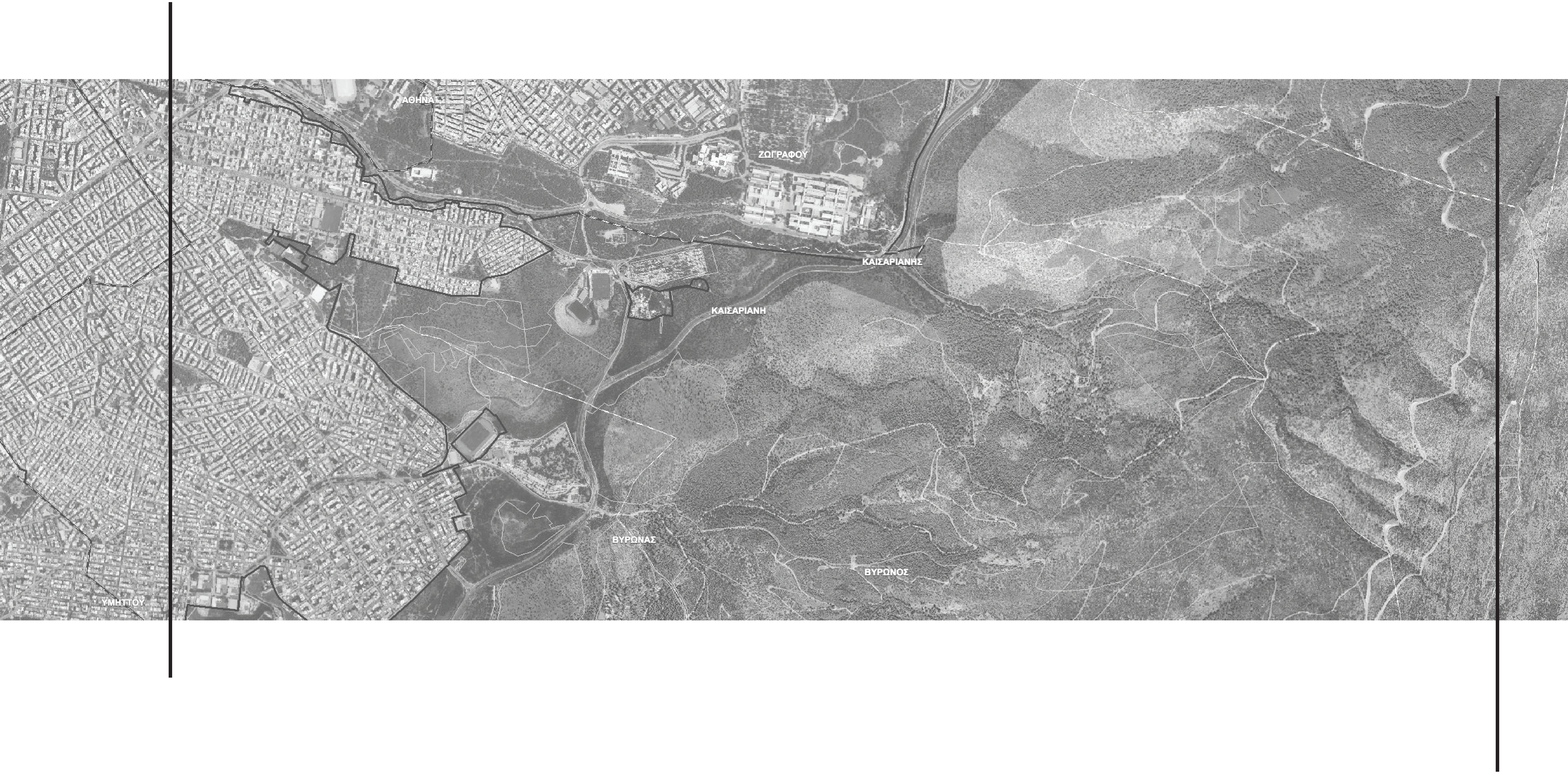

Network of laboratories
Civic technologies co-production

Workshop: Co-designing digital infrastructures

thematic aera : **Supporting forest protection teams of Municipality of Kesariani**

B1. meteorological station design canvas

need covered

expected functionalities and capacities

technical requirements of installation and functioning

potential institutions which can offer resources or knowledge in development and sustainability

basic problems or limitations

station functioning diagram

dangers and vulnearable points

workshop design and implementation

OPEN LAB ATHENS

with the support of

ΔΗΜΟΣ ΚΑΙΣΑΡΙΑΝΗΣ

Network of laboratories
Civic technologies co-production

Workshop: Co-designing digital infrastructures

thematic aera : Supporting forest protection teams of Municipality of Kesariani

B2. coordination platform design canvas

mapping of needs from canvas A	analysis of needs' basic characteristics	diagram of ongoing process	design of new process with the use of digital tool (workflow diagram)	individuals/institutions that are involved or affected

workshop design and implementation
OPEN LAB ATHENS

with the support of
ΔΗΜΟΣ ΚΑΙΣΑΡΙΑΝΗΣ
